# Practice of novel method of bedside postpyloric tube placement in patients with coronavirus disease 2019

**DOI:** 10.1186/s13054-020-02863-0

**Published:** 2020-04-07

**Authors:** Shou-Tao Yuan, Wen-Hao Zhang, Lei Zou, Jia-Kui Sun, Ying Liu, Qian-Kun Shi

**Affiliations:** 1 Department of Intensive Care Unit, Nanjing First Hospital, Nanjing Medical University, 68 Changle Road, Nanjing, 210006 Jiangsu Province China; 2 Department of Isolation Unit E1-5F, Tongji Hospital, Huazhong University of Science and Technology, Wuhan, Hubei Province China

During our clinical work against the epidemic of coronavirus disease 2019 (COVID-19) in Wuhan [1], we observed a high incidence of malnutrition in critically ill patients (data unpublished). Therefore, nutritional therapy was very important. In patients with dysphagia and a very high aspiration risk, postpyloric enteral nutrition (EN) was required [2]. However, how to place the postpyloric tube was a challenge in COVID-19 patients. Patients with masks removed (to expose the nasal cavity) were seriously infectious to doctors. Besides, it was difficult to perform the tube placement bedside for doctors with heavy medical protective clothes, goggles, and face shield. Here, we shared our practice of novel placing method in Wuhan.

A 130-cm-long non-spiral transpyloric tube with a guide wire (CH10-130, inner diameter 2.0–2.1 mm, Flocare, Nutricia Ltd., Wuxi, China) (Fig. 1a) was used in our isolation unit. The procedure of placement was similar to the method reported by our previous study [3, 4]. Patients were placed in right decubitus position about 30–45° with bed head raised at about 30°. After esophageal placement and gastric placement, the postpyloric placement was performed by advancing the tube at 5–10 cm intervals gradually and checking its tip position each time. Subsequently, the tip position would be confirmed by abdominal plain radiographs or gastrointestinal ultrasound bedside. The tube that we used has several advantages compared with spiral tube. First, the price of Flocare tube (approximately $22) is 1/3 less compared with spiral tube (approximately $71) in China. Second, the Flocare tube has two side holes near its tip (Fig. 1b); it is less likely to be blocked. Third, the guide wire is shorter in length compared with the tube; therefore, the rigid tip could not damage the digestive tract during our placing procedure

**Fig. 1:**
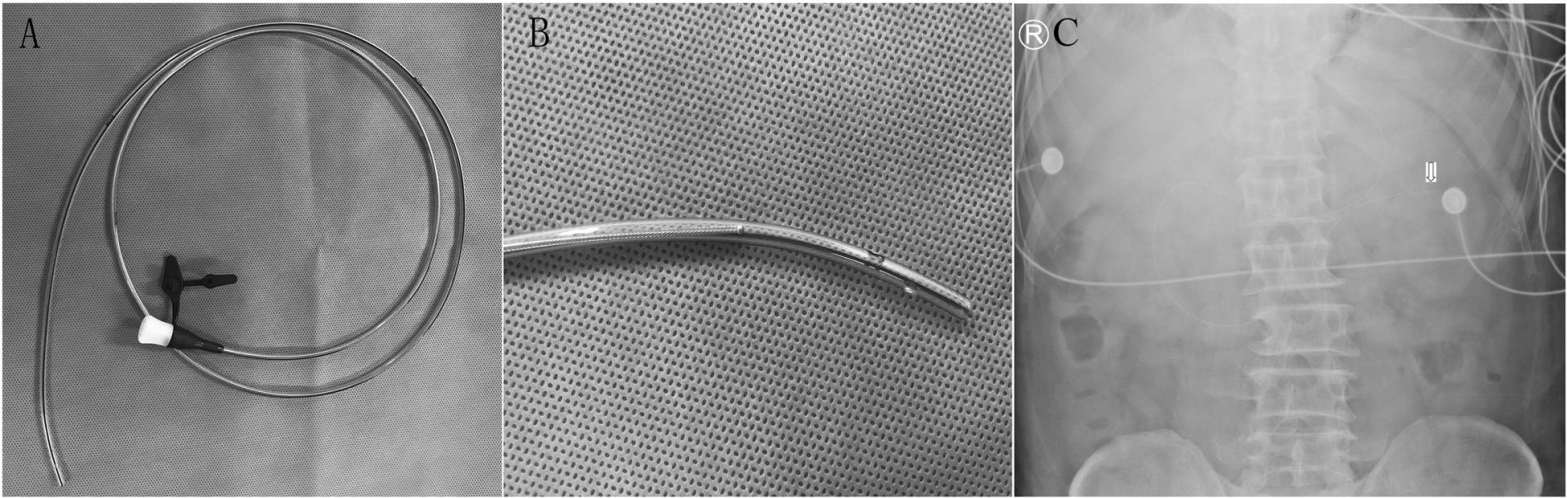
The 130-cm-long transpyloric tube with a guide wire (CH10-130, inner diameter 2.0–2.1 mm, Flocare, Nutricia Ltd., Wuxi, China) used in our unit (**a**). This Flocare tube has two side holes near its tip (**b**). Abdominal plain radiograph showed the tip of Flocare tube was positioned near the Treitz ligament (**c**)

There have been three patients who received our novel method of postpyloric tube placement. The 3 cases were all successful at the first attempt (Fig. 1c). The median time of procedure was 19 (14–25) minutes, and the median insertion length was 105 (95–110) cm. No operation- and tube-related complications were found. Considering the less expensive tube and high success rate, our novel blind bedside postpyloric placement may be easier to perform in patients with COVID-19 worldwide.

## Data Availability

Not applicable.
